# Diffuse Large B‐Cell Lymphoma Arising in Postpneumonectomy Cavities: Report of Two Cases and Literature Review

**DOI:** 10.1155/carm/2936999

**Published:** 2026-06-19

**Authors:** Patricia Capdevila, Elisa Gómez, Cristobal Carrasco, Carlos Escrivá, Emilio Soria, Josep M. Esteve, Daniel Vidal, Alba Ortiz, Javier Caballero, Jose Gomez-Codina

**Affiliations:** ^1^ Department of Medical Oncology, Hospital Universitario y Politécnico La Fe, Valencia, Spain, hospital-lafe.com

## Abstract

Diffuse large B‐cell lymphoma (DLBCL) most commonly arises in nodal regions but may also present extranodally. We report two cases of large B‐cell lymphoma developing in postpneumonectomy cavities more than a decade after surgery for primary lung carcinoma. In both patients, no history of systemic immunodeficiency, organ transplantation, or chronic immunosuppressive therapy was present, and HIV, hepatitis B, and hepatitis C serologies were negative. In Case 1, the lymphoma showed an activated postgerminal center phenotype and was negative for EBER and HHV‐8. In Case 2, the tumor was CD20‐negative and EBER‐positive, with pathological features of inflammation‐associated large B‐cell lymphoma. PET‐CT was used for staging and response assessment in both patients, and both achieved complete metabolic remission after curative‐intent chemotherapy. These cases underscore a rare clinical entity that differs pathophysiologically from pyothorax‐associated lymphoma (PAL) or primary effusion lymphoma (PEL). They highlight the postpneumonectomy cavity as a rare but potentially permissive microenvironment for large B‐cell lymphomagenesis and underscore the biological heterogeneity of these presentations.

## 1. Introduction

Diffuse large B‐cell lymphoma (DLBCL) accounts for approximately 30%–40% of non‐Hodgkin lymphoma cases and typically presents with rapidly enlarging nodal masses [1]. Extranodal involvement is seen in nearly one‐third of cases, with common sites including the gastrointestinal tract, central nervous system, bone marrow, and skin. Thoracic presentations are less frequent and generally associated with specific contexts, such as immunosuppression or chronic inflammation. Pleural or thoracic cavity involvement is rare and, when present, typically aligns with distinct clinicopathological entities such as pyothorax‐associated lymphoma (PAL) or primary effusion lymphoma (PEL) [2, 3].

Among the thoracic presentations, PAL is a well‐described subtype associated with a clinical history of longstanding pyothorax or chronic inflammation of the pleura resulting from therapeutic artificial pneumothorax for the treatment of pulmonary tuberculosis or tuberculous pleuritis. These lymphomas are usually Epstein–Barr virus (EBV)‐positive, B‐cell in origin, and associated with systemic inflammatory signs [2]. Alternatively, PEL is a distinct HHV‐8‐related LBCL, characteristically presenting as malignant effusions without tumor masses in immunocompromised individuals [3].

Recently, fluid overload‐associated LBCL (FO‐LBCL) has been described as a distinct clinicopathological entity occurring predominantly in elderly, immunocompetent patients. These lymphomas typically present in anatomical sites exposed to chronic fluid accumulation or mechanical stress, such as serous cavities or spaces altered by prior surgery. Unlike pyothorax‐associated or HHV‐8‐related lymphomas, FO‐LBCL is characteristically negative for EBV and HHV‐8 and lacks a background of overt inflammation or immunodeficiency [4].

We report two rare cases of DLBCL presenting as pleural‐based masses in postpneumonectomy cavities, unrelated to chronic pyothorax or viral infection. We hypothesize that surgically altered, chronically remodeled thoracic spaces may represent permissive environments for lymphoid neoplasia, even in the absence of chronic infection or inflammation.

## 2. Case Reports

### 2.1. Case 1

A 66‐year‐old male was initially diagnosed in December 2003 with a Stage IIA (pT2N0M0) right lung adenocarcinoma. He underwent a right pneumonectomy with curative intent, with no adjuvant therapy. In May 2006, a mediastinal recurrence was detected and managed with concurrent chemoradiotherapy (carboplatin‐paclitaxel and thoracic radiotherapy), resulting in a complete response. A second primary lesion in the contralateral lung was detected in October 2013 and treated with stereotactic body radiotherapy (SBRT) to 60 Gy, achieving complete response.

In April 2017, during routine surveillance imaging, a new solid lesion measuring 23 × 41 mm was detected in the right postpneumonectomy cavity (Figure 1). The patient was asymptomatic, with preserved performance status and no B symptoms. CT‐guided core needle biopsy of the pleural‐based lesion demonstrated DLBCL with an activated postgerminal center phenotype. Immunohistochemistry showed strong expression of CD20, BCL6, MUM1, and BCL2, without expression of CD30, CD5, or HHV‐8. EBER in situ hybridization was negative, and the Ki‐67 proliferation index was approximately 95%. FISH studies for MYC and BCL2 rearrangements were negative.

**FIGURE 1:**
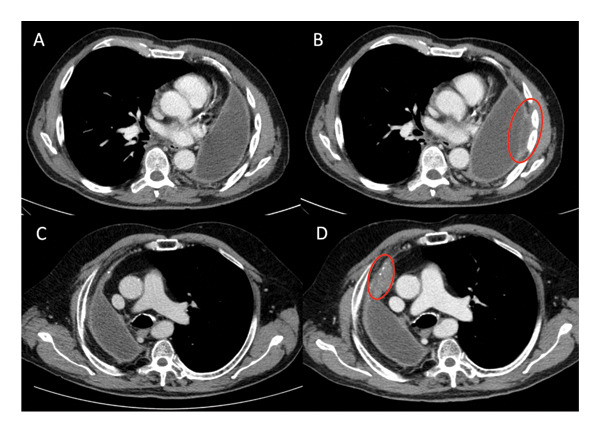
(A) Routine thoracoabdominal CT (09/2016): right postpneumonectomy cavity with calcifications, no significant changes. (B) Follow‐up CT (04/2017): emergence of a solid anterior lesion within the postpneumonectomy cavity, measuring 23 × 41 mm. (C) Routine CT (12/2022): stable postpneumonectomy cavity. (D) Follow‐up CT (06/2023): elongated necrotic mass extending from the cavity wall into the extrapleural fat, abutting the lateral arcs of the 5th and 6th ribs without evidence of bone destruction; lesion size approximately 57 × 13 × 39 mm.

Baseline laboratory studies were unremarkable, including LDH. Serologic testing for HIV, hepatitis B, and hepatitis C was negative. Additional immune work‐up showed normal immunoelectrophoresis and a CD4+ T‐cell count of 415 cells/μL. The patient had no history of organ transplantation, known immunodeficiency, or chronic immunosuppressive therapy. Bone marrow aspirate and biopsy showed no evidence of lymphoma infiltration.

Baseline PET‐CT (June 13, 2017) showed an intensely hypermetabolic mass in the anterior aspect of the postpneumonectomy cavity (SUVmax 16.8), with additional hypermetabolic nodules in the right extrapleural fat and right hemidiaphragm, as well as a small hypermetabolic periesophageal lymph node. No infradiaphragmatic or osseous lymphoma involvement was identified. The patient was diagnosed with stage IV‐A DLBCL arising in the postpneumonectomy cavity (IPI 2) and received six cycles of R‐CHOP every 21 days. Serial imaging demonstrated an excellent response to therapy, culminating in complete radiological remission (Figure 2). End‐of‐treatment PET‐CT (November 14, 2017) demonstrated a complete metabolic response, with the disappearance of the previously described hypermetabolic lesions. He remained alive and disease‐free at the last follow‐up in October 2025.

**FIGURE 2:**
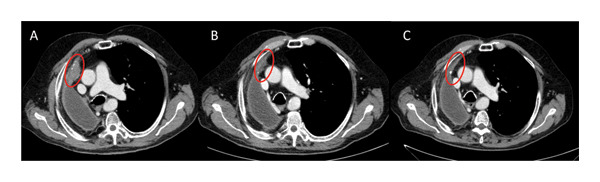
(A) Initial thoracoabdominal CT (04/2017): solid mass measuring 23 × 41 mm within the postpneumonectomy cavity. (B) Partial response after three cycles of chemotherapy (08/2017), with reduction in lesion size. (C) Complete radiological response after six cycles of chemotherapy (11/2017).

### 2.2. Case 2

A 78‐year‐old male was diagnosed in June 2003 with Stage IIB (pT2N1M0) squamous cell carcinoma of the right lung and underwent a right pneumonectomy without adjuvant therapy. In August 2005, he experienced a local recurrence managed with concurrent chemoradiation (carboplatin‐paclitaxel and thoracic radiotherapy), achieving sustained remission. He remained asymptomatic and free of recurrence on surveillance imaging for nearly 18 years.

In June 2020, the second patient developed chest pain and constitutional symptoms. CT imaging revealed two contiguous left chest wall masses arising adjacent to the postpneumonectomy cavity, the largest measuring 57 mm, with involvement of the lateral 7th rib and an additional implant in the left costophrenic fat (Figure 1). PET‐CT confirmed two intensely hypermetabolic lesions in the left chest wall (SUVmax 24.8 and 17.2, respectively), without convincing evidence of distant lymphoma involvement. Mildly hypermetabolic nodes in the extrapleural fat, internal mammary chain, and interaortocaval region were considered unlikely to be tumoral because they were morphologically stable compared with prior imaging.

Ultrasound‐guided core needle biopsy of the 7th left costal arch mass showed a LBCL with dense fibrous stroma, geographic necrosis, and an inflammatory background. The neoplastic cells were positive for CD45, PAX5, CD79a, MUM1, CD30, BCL2 (> 50%), and c‐MYC (> 40%), and negative for CD20, CD3, CD5, CD4, CD8, ALK, and EMA. BCL6 expression was limited (approximately 10%). The Ki‐67 proliferation index was approximately 90%. EBER in situ hybridization was positive. FISH studies showed no rearrangements involving MYC, BCL2, or BCL6, supporting a double‐expressor but not double‐hit phenotype.

Serologic testing for HIV, hepatitis B, and hepatitis C was negative. The patient had no history of organ transplantation or chronic immunosuppressive therapy. Based on the clinical, radiologic, and pathologic findings, the diagnosis was EBV‐positive inflammation‐associated LBCL arising in a chronically remodeled postpneumonectomy cavity. Staging was consistent with Lugano stage IE extranodal disease, with low‐risk IPI. The patient received six cycles of CHOP‐14 from July to September 2020. End‐of‐treatment PET‐CT (November 13, 2020) showed marked regression of both lesions, with only minimal residual tissue and metabolic activity below hepatic uptake, consistent with complete metabolic response (Deauville score 2). He remains in complete remission on follow‐up.

## 3. Discussion

LBCL arising in postpneumonectomy cavities is exceptionally uncommon. According to the current WHO classification, LBCL arising in extranodal sites comprises a biologically heterogeneous group, and their interpretation requires integration of morphology, immunophenotype, viral status, and clinical context [1]. In the specific setting of a postpneumonectomy cavity, only isolated cases have been reported, highlighting the rarity of this presentation. Riehl et al. described a malignant non‐Hodgkin lymphoma developing years after pneumonectomy, suggesting that a chronically altered postsurgical thoracic cavity may, in rare circumstances, become a permissive site for lymphoid neoplasia [5].

The differential diagnosis includes PAL, an LBCL classically arising in the pleural cavity after long‐standing pyothorax or chronic pleural inflammation, often in association with EBV [2]. This entity supports the concept that chronic inflammation may drive lymphomagenesis in restricted pleural spaces. However, neither of our patients had a history of chronic pyothorax, which argues against classic PAL despite the shared feature of lymphoma arising in a chronically remodeled pleural environment [2].

PEL is also an important consideration, but its clinicopathologic profile differs substantially from our cases. PEL is typically associated with HHV‐8 infection, often develops in immunocompromised patients, and usually presents as lymphomatous effusions without a dominant solid mass [3]. By contrast, both of our patients had negative serologies, no history of transplantation or chronic immunosuppressive therapy, and presented with solid pleural/chest wall lesions rather than isolated serous effusions, making PEL unlikely [3].

From a biological perspective, our two cases should not be regarded as a single homogeneous entity. Case 1 showed an activated postgerminal center phenotype, with negative EBER and HHV‐8 studies, in the absence of clinical or laboratory evidence of systemic immunodeficiency. In contrast, Case 2 was EBER‐positive and showed fibrosis, necrosis, and an inflammatory background, findings more consistent with an inflammation‐associated LBCL arising in a chronically altered thoracic space. In this regard, recently described FO‐LBCL and other cavity‐related EBV‐associated large B‐cell proliferations support the concept that confined anatomical spaces with chronic fluid, fibrin deposition, or persistent inflammation may provide a permissive microenvironment for lymphomagenesis [4]. Although our second case does not fit perfectly within those categories, it likely belongs to the same broader biological spectrum of LBCL arising in chronically remodeled local environments [1, 4].

Additional support for this inflammation‐related mechanism comes from other unusual extranodal LBCL arising in chronically damaged tissues. Fujimoto et al. reported an EBV‐associated postsurgical DLBCL adjacent to prosthetic material, reinforcing the possible role of long‐term local immune dysregulation and chronic tissue remodeling in lymphoid transformation [6]. Likewise, chest wall DLBCL developing many years after prior trauma has also been described, again suggesting that longstanding local tissue injury may contribute to lymphomagenesis in susceptible settings [7].

Taken together, these observations support the hypothesis that postpneumonectomy cavities may function as a chronic inflammatory and immunologically altered niche in which LBCL may rarely develop. Prior thoracic surgery, fibrosis, altered lymphatic drainage, previous radiotherapy, and persistent local antigenic stimulation may all contribute to this process. Clinically, these cases underline the importance of considering lymphoma in the differential diagnosis of newly detected pleural or chest wall masses arising in postpneumonectomy cavities, even many years after lung cancer treatment, because prompt biopsy, appropriate staging with PET‐CT, and timely curative‐intent chemotherapy may lead to excellent outcomes [5].

## 4. Conclusion

LBCL arising in postpneumonectomy cavities is exceptionally rare and likely biologically heterogeneous. These cases highlight that a chronically remodeled pleural space may provide a permissive microenvironment for lymphomagenesis, even many years after lung cancer surgery. Clinicians should therefore consider lymphoma in the differential diagnosis of new pleural or chest wall masses arising in postpneumonectomy cavities, including in long‐term cancer survivors without overt immunodeficiency. Early histologic diagnosis and curative‐intent chemotherapy may lead to durable complete remissions, even in anatomically complex thoracic locations.

## Author Contributions

All authors contributed to the conception and design of the work. Patricia Capdevila drafted the manuscript. Elisa Gómez, Cristobal Carrasco, Carlos Escrivá, Emilio Soria, Josep M. Esteve, Daniel Vidal and Alba Ortiz contributed to data collection and interpretation. Jose Gomez‐Codina and Javier Caballero supervised the project and provided critical revision of the manuscript.

## Funding

The authors received no specific funding for this work.

## Disclosure

All authors approved the final version.

## Consent

Written informed consent was obtained from the patients for publication of the case details and associated images.

## Conflicts of Interest

The authors declare no conflicts of interest.

## Data Availability

All data supporting the findings of this study are included within the article. Further inquiries can be directed to the corresponding author.
